# Effects of Different Shelling Methods on Data Variability during Field Screening for Reduced Aflatoxin Contamination in Maize

**DOI:** 10.3390/toxins16070324

**Published:** 2024-07-19

**Authors:** Alison Adams, Daniel Jeffers, Shien Lu, Baozhu Guo, W. Paul Williams, Jake C. Fountain

**Affiliations:** 1 Department of Plant Pathology, University of Georgia, Griffin, GA 30223, USA; 2 USDA-ARS, Corn Host Resistance Research Unit, Mississippi State, MS 39762, USA; 3 Department of Biochemistry, Molecular Biology, Entomology, and Plant Pathology, Mississippi State University, Mississippi State, MS 39762, USA; 4 USDA-ARS, Crop Genetics and Breeding Research Unit, Tifton, GA 31793, USA

**Keywords:** *Aspergillus flavus*, aflatoxin, germplasm screening, variation, maize breeding

## Abstract

Non-genetic variation limits the identification of novel maize germplasm with genetic markers for reduced *Aspergillus flavus* infection and aflatoxin contamination. Aflatoxin measurements can vary substantially within fields containing the same germplasm following inoculation with *A. flavus*. While some variation is expected due to microenvironmental differences, components of field screening methodologies may also contribute to variability in collected data. Therefore, the objective of this study is to test the effects of three different shelling methods (whole ear (WE), ear end removal (EER), and inoculation site-surrounding (ISS)) to obtain bulk samples from maize on aflatoxin measurements. Five ears per row of three inbred lines and two hybrids were inoculated with *A. flavus*, then shelled using the three different methods, and aflatoxin was quantified. Overall, EER and ISS resulted in reduced coefficients of variance (CVs) in comparison to WE for both inbred and hybrid maize lines, with two exceptions. Susceptible B73 showed increased CVs with both EER and ISS compared to WE, and resistant Mp719’s EER CVs marginally increased compared to WE. While WE is the standard practice for most breeding programs due to its technical simplicity, EER and ISS may allow for finely phenotyping parental lines for further breeding applications.

## 1. Introduction

The development and release of maize germplasm with consistently reduced aflatoxin contamination and infection by *Aspergillus flavus* is a continuing goal for the aflatoxin research community. The development of such germplasm would remedy some of the annual billion-dollar global economic losses due to aflatoxin contamination in maize and progress has already been made [1,2]. However, the production of lines with consistently reduced aflatoxin contamination has proven elusive. While several host resistance mechanisms have been proposed to contribute to reducing aflatoxin contamination and fungal colonization, efforts in developing selectable genetic markers and narrowing/prioritizing these mechanisms for selection has proven difficult.

Aflatoxin contamination is notoriously variable when comparing different germplasm, seasons, and geographical locations. This high level of variance is detrimental to the identification of germplasm with consistently low aflatoxin contamination that would be useful in variety development. Variance in aflatoxin data due to genotype × environment (G × E) interactions or other sources, such as the analytical methods used to measure aflatoxin, can hinder genetic mapping studies [3,4]. The difficulty in mapping genetic markers consistently associated with reduced aflatoxin can be seen in relatively recent studies. For example, in Womack et al., a quantitative trait locus (QTL) analysis was performed using an F2:3 mapping population (241 individual families) derived from a cross between Mp705 × Mp719 (susceptible × resistant) [1]. This study identified several QTLs that ranged in percent variance explained (PVE) from 3.3 to 15.4% and QTLs consistent across environments at bins 1.06, 1.07, and 3.09 exhibited a combined PVE of 24.6%. Similar results have been obtained for other aflatoxin QTL studies in corn [5,6,7,8]. While potentially informative of the mechanisms involved in host resistance, markers with these low PVEs are more difficult to adopt in breeding programs due to needing to simultaneously genotype multiple markers, though genotyping technologies are improving to allow this. Overall, these studies clearly demonstrate the challenges faced by breeders in the identification of genetic markers useful for marker-assisted selection (MAS) for reduced aflatoxin, i.e., the highly quantitative nature of resistance and variation in phenotypic data.

In mapping studies, germplasm field screening experiments have high observable degrees of variation within the same season and field, and even among replicated plots. For example, in recent screens examining aflatoxin accumulation in diverse inbreds and topcross F1 hybrids, a high coefficient of variance (CV) could be observed within each line, even among replicates within the same season/environment [9]. This limited the statistical power of post-hoc analyses to identify significant differences in aflatoxin levels between germplasm. Similar issues with plot-to-plot variability within the same season have also been faced by other screening studies [10]. During the execution of these studies, phenotyping methods are relatively straightforward: inoculation of lines at mid-silk, harvesting ears at maturity, shelling of inoculated ears (typically in bulk by rows or plots), grinding and subsampling, and aflatoxin quantification [11].

Sampling methods are fairly standardized across most breeding studies where whole inoculated ears from within a plot or row are bulk shelled [12]. This method is useful because it is technically simple, rapid, and not labor intensive when performed with electric shellers and grinders. However, there is the possibility that bulk shelling may introduce unintended variation and bias in screening studies through dilution effects. For example, when comparing lines, particularly inbred germplasm, there is likely to be variation in ear size and overall mass of kernels (Figure S1). If the inoculation method is consistent, then a similar number of kernels will be inoculated on each ear, resulting in a lesser proportion of inoculated kernels in ears with higher kernel mass, thus diluting aflatoxin measurements taken from bulk sampling. The opposite would also hold true in low kernel mass ears, resulting in inflated aflatoxin levels. Dilution effects may also result from variation in yield and ear filling, both of which can be influenced by the environment [13,14] and may result in plot-wise or seasonal variation in data. There is also the possibility of secondary *A. flavus* infections or insect damage, particularly on the tips of ears, which may also affect aflatoxin measurements when examining whole ears [15].

**Figure 1:**
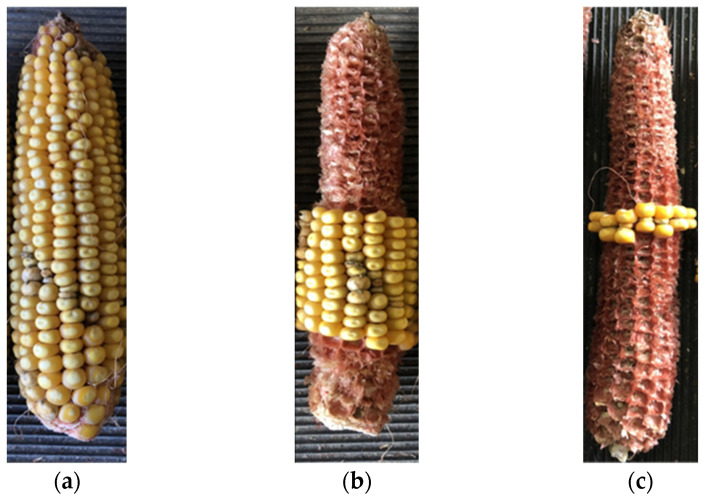
Representative images of the three shelling methods used in this study: whole ear (WE) shelling (**a**), ear end removal (EER) shelling (**b**), and inoculation site-surrounding (ISS) shelling (**c**).

Given the need for greater reproducibility in aflatoxin data for the development and utilization of newer genetic mapping populations, and the screening of candidate germplasm in breeding programs, here we sought to explore the use of different shelling methods to yield more consistent aflatoxin measurements (i.e., low CVs) for field screening experiments (Figure 1). Such a method, if technically straightforward, low cost, and effective, may result in adoption by breeding programs to improve genetic gains for mitigating aflatoxin contamination through germplasm development, as well as finer genetic mapping and selectable marker identification.

## 2. Results and Discussion

Examination of aflatoxin contamination in different germplasm is an important initial phase in breeding programs to identify candidate resistant lines for use in genetic mapping and novel variety development. However, excessive variation in aflatoxin data in maize field experiments is a major limiting factor in the identification of significant marker-trait associations and the accurate evaluation of individual lines for resistance to aflatoxin contamination. To counter this, here we evaluated shelling methodology as one potential variance component of the process of collecting aflatoxin data. Three different shelling methods were evaluated using both inbred and hybrid maize in a single field season with extensive replication to see the effects on overall data variance each method would have. In addition, this experiment sought to provide information on the level of aflatoxin contamination occurring in the in-development inbred line SynAM1 P43 and evaluate the heritability of its reduced aflatoxin traits into F1 hybrids. The commercial hybrid DKC 67-44 was also examined in Mississippi field conditions for aflatoxin contamination levels post-inoculation since it is a commonly utilized dry land variety [16].

### 2.1. Effect of Shelling Method on Aflatoxin Measurement Variability

The raw aflatoxin measurements for the experiment ranged from 22 µg/kg to as high as 22,000 µg/kg (File S1, Figure S2). Therefore, all the inoculated samples examined in the study, regardless of line and shelling methods, exceeded the current regulatory limit of 20 µg/kg for total aflatoxin content set by the US Food and Drug Administration (FDA) [17]. Examination of the distribution of this raw aflatoxin data revealed that it was heavily skewed, necessitating a log_2_(x + 1) transformation to satisfy conditions of normality for further statistical analyses (Figure S3). Analysis of variance of the transformed data showed significant line effects (*p* < 2 × 10^−16^) and significant line × treatment interaction effects (*p* = 6.71 × 10^−9^) with insignificant treatment effects (*p* = 0.1520) (Table 1). This indicates that while the shelling methods had similar ranges of aflatoxin measurements when comparing between them directly, there is a significant difference in aflatoxin measurements both between the lines used in the study and a significant effect of shelling method on the measurements obtained for individual lines.

In most field trials related to aflatoxin research, whole ear (WE) shelling is the principal method employed [11,12]. Here, the selected inbred lines performed as expected. The resistant control Mp719 showed the lowest level of raw aflatoxin with an average of 84 ± 50 µg/kg. B73, the susceptible check, exhibited the highest levels of aflatoxin contamination with 12,000 ± 2062 µg/kg (Table 2, File S1, Figure S2). The unreleased inbred line SynAM1 P43 was intermediate between these checks with 774 ± 733 µg/kg. For the hybrids, SynAM1 P43 × B73 at 1448 ± 1100 µg/kg was not significantly different from the commercial hybrid DKC 67-44 at 2112 ± 1300 µg/kg. The aflatoxin contamination observed in the F1 was also not significantly different from the parental inbred SynAm1 P43 but was significantly less than that observed for B73, suggesting that the moderate level of resistance observed in SynAM1 P43 is heritable and effective in reducing aflatoxin contamination (Table 2). The levels of aflatoxin observed here are comparable to previous studies [9] using WE shelling which showed 1406 ± 1654 µg/kg and 731 ± 807 µg/kg for SynAM1 P43 in 2014 and 2015 trials in Georgia, respectively. SynAM1 P43 × B73 also showed comparable levels in the previous trial in 2016 in Georgia (1151 ± 502 µg/kg). B73, however, showed much higher levels of contamination in the present study compared to the previous study in Georgia where measurements of 1824 ± 1091 and 2170 ± 519 µg/kg were made in 2014 and 2015, respectively. Inbred line Mp719 was also comparable to a previous multi-year trial in Texas with bulk shelling log_10_(x + 1) transformed aflatoxin levels reported as 1.4 µg/kg [18]. Meanwhile, SynAM1 P43 was reported to be much lower at 1.5 µg/kg. The higher overall aflatoxin levels observed in the present study in comparison to these earlier results is most likely due to the use of different *A. flavus* isolates between these studies. The previous studies [9] used the less toxigenic strain, NRRL3357, while the present study used the more toxigenic strain, AF13. The Texas study scattered *A. flavus*-inoculated maize kernels while the Georgia studies used direct-to-ear inoculation.

When considering different shelling methods, for the first new method, ear end removal (EER) shelling, the rationale was to eliminate the possibility of variance resulting from two potential sources. The first was the possibility of variation in ear tip filling between plots due to variation in field microenvironmental differences between plots. The second was the potential for secondary *A. flavus* infections either naturally through the end of the ear and the silk channel or because of insect injury on the ear tip (particularly armyworms and ear worms), and/or pooled primary or secondary inoculum at the base of the ear in the husk (Figure S1, white box). This is particularly of concern in hybrid corn where short-husk ears have been recently observed in newly released hybrids where ear tips protrude beyond the husk, exposing the ear tip to the environment in response to various stresses [19]. This could result in increased risk of insect damage and mycotoxin contamination. However, overall aflatoxin levels detected between WE and EER shelling were not significantly different (Table 3).

For the second new method, inoculation site-surrounding (ISS) shelling, the goal was to examine only kernels immediately surrounding the inoculation point on the ear (Figure 1). The rational was that overall aflatoxin accumulation in these surrounding kernels should correlate with the actual level of overall resistance of the examined line to either *A. flavus* growth or to aflatoxin contamination. Overall, ISS shelling resulted in significantly different measurements compared to WE and EER shelling, though the general trends of the line relationships were preserved (Table 2). For the inbreds, B73 had significantly less aflatoxin with 3440 ± 2322 µg/kg compared to data obtained from the other methods, though it remained the most susceptible of the examined lines. However, B73 was not significantly different from DKC 67-44 and SynAM1 P43 which had 2194 ± 930 µg/kg and 1188 ± 267 µg/kg, respectively. Mp719 significantly increased with ISS shelling compared to the other methods, with 559 ± 269 µg/kg (Table 2).

Comparison of the variance between these shelling methods was the primary goal of this experiment. To that end, coefficients of variance (CVs) were calculated for each line × shelling method combination to determine whether there was an effect of shelling method on overall variance (Table 2 and Table 3). Overall, there were significant differences when examining line, shelling method, and their interactions, more so when examining the transformed aflatoxin data compared to the raw data. Examining the effects of the different shelling methods on a line-by-line basis, the susceptible B73 had the highest CV for ISS but the lowest CV for WE, whereas the more resistant Mp719 had neither the highest nor lowest CVs for any shelling method. However, based on a modified SLRT (Table 3), the data variation in the aflatoxin measurements of Mp719 and DKC 67-44 did not change significantly across shelling methods. For the lines B73, SynAM1 P43, and SynAM1 P43 × B73, the shelling method significantly influenced the data variation. B73′s CV values increased as less kernels were used in the shelling method, whereas the CVs for both SynAM1 P43 and SynAM1 P43 × B73 decreased. The CV trends seen in the F1 hybrid SynAM1 P43 × B73 were more like those of its resistant parent, further indicating an intermediate resistance trait (Table 2). Interestingly, SynAM1 P43 had the highest CV with WE and EER shelling, but the lowest in ISS shelling. Through observing the raw data, it appears that when extreme outliers are removed, additional outliers are revealed for this and two other lines and correlate to the increase in CV values (Figure S3). For SynAM1 P43, while this is most likely due to typical variation in aflatoxin data observed in inbred maize due to genotype × environment interactions, variance in anther color was seen in this line in the field. This may be indicative of ongoing genetic segregation or impurity in the seed stocks for this line, which may contribute to the observed variance in aflatoxin data and may warrant investigation prior to official germplasm release.

### 2.2. Observations from Shelled Kernel Mass, Ear Length, and Ear Fill Percentage

The three shelling methods used in this study led to significantly different average weights (Table S1). This result, however, was expected as the shelling methods were designed to capture different proportions of kernels from an ear. The mean of the EER and ISS weights for each line were 25.2–41% and 3–6%, respectively, of the mean of WE weights. Additionally, the two hybrids had significantly higher weights compared to the three inbreds for WE shelling. In comparison to the aflatoxin measurements, the CVs for shelled kernel mass were all lower, except B73′s CV for WE. For B73, whose kernels are loosely attached to the ear and were more prone to falling off without much force, the highest CVs (shelled kernel mass and aflatoxin) were both seen with ISS. This highlights a potential limitation of ISS shelling methods for highly susceptible lines, particularly those with loose kernel attachment as seen with B73. The inadvertent loss of kernels during sample handling would likely have negligible effects for WE or EER shelling due to their larger sample masses. However, for ISS, given its small sample size, the loss of a single kernel from the sample during handling or shelling could contribute to increasing variance in final aflatoxin measurements. For SynAM1 P43, the CVs for ISS were within 0.05. For Mp719, the lowest CVs were both seen in WE and for DKC 67-44, the lowest CVs were both seen in EER. Further statistical analysis on the significant interaction between line and treatment revealed inbreds have a higher degree of variability when compared to hybrids, further indicating the need to fine phenotype potential inbred parentals for hybrid crosses (Table S2). Overall, this limited data set indicates kernel retention during sampling and/or shelling can be a key step to reducing variability in collected aflatoxin data.

We also investigated whether the density of kernels on the ear had an impact on the quality of shelling data. Environmental damage may lead to kernel loss at the tips, shortening the percent of the total ear that is filled and potentially skewing results. Understandably, an ANOVA did show that ear length and fill significantly differed by line (Tables S3 and S4). There was no significant difference when considering line by treatment, indicating the treatment effect seen in ear length could be an artifact of the line and not the treatment itself. For both, there was a slight difference among replicates within a treatment plot of the same line; however, as mentioned earlier, total ear length naturally varies.

**Figure 2:**
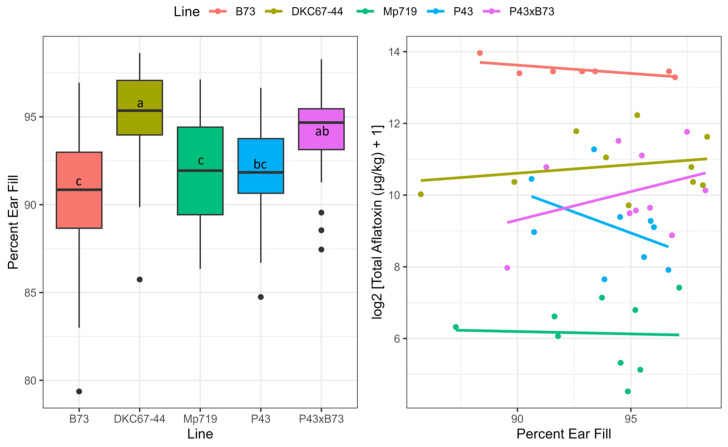
Boxplots of percent ear fill across all ears collected for each line (**left**). Dot plot with regression lines of percent ear fill and log_2_(x + 1) aflatoxin measurements from WE shelling only (**right**). Measurements within a trait possessing the same letter labels were not significantly different based on Tukey’s post-hoc analysis (α = 0.05). The “SynAM1” of P43 was removed for simplicity.

With respect to ear fill, the hybrids expectedly had the highest fill percentage means (94–95%), while the inbred B73 had the lowest (90%) (Figure 2, Table S3). Although there were no significant associations between ear fill and aflatoxin measurements from WE shelling, the inbreds appear to have less aflatoxin as their ear fill increases, whereas the hybrids have more (Figure 2). A possible explanation for the inbreds is that a more filled ear percent indicates less environmental damage at the tip of the ears (i.e., lower chance of secondary infections). A possible explanation for the hybrids is that, due to the enhancements of breeding, the ear is so large that it pokes out of the top of the husk and exposes the kernels at the tip to environmental damage (i.e., higher chance of secondary infection) [19,21].

### 2.3. Comparison to Other Sampling Methods and Mycotoxin-Related Studies

Often, maize shelling studies are technical studies that evaluate the impact of new low-cost equipment or alternative manual techniques on yield, labor, and resources, especially for traditional, small-scale farms [22]. In terms of mycotoxin research, there has been great focus on developing sampling, sorting, and shelling methods to reduce contamination in cash crops before being sold to industry and/or consumers [23,24,25]. A great deal of research has also been performed on examining the effects of using different *A. flavus* inoculation techniques for variety evaluation in breeding programs [26]. A majority of inoculation and maize variety evaluation studies, however, then proceed to bulk the harvested ears in a plot or subplot for shelling followed by grinding and subsampling of this material for aflatoxin analysis [12,27]. Some studies have also examined individual ears or subsets of kernels when evaluating specific hypotheses, but not commonly for larger scale germplasm evaluations [28]. Thus far, however, no other experiment has been conducted to examine different shelling techniques for aflatoxin contamination phenotyping of maize breeding population or germplasm screening in the field to reduce data variance.

Overall sample weight is another potential component of variance, particularly when working with maize with higher levels of aflatoxin contamination. For example, Johansson et al. (2001) found that overall data variance increased as measured aflatoxin concentration increased, and that that variance was derived mainly from sampling compared to sample processing and aflatoxin analyses [29]. They also found that examining larger samples of grain had lower coefficients of variance compared to smaller ones (5.00 kg samples divided into 100 g subsamples for analysis vs. 1.13 kg divided into 50 g subsamples) at the same estimated aflatoxin concentration (20 µg/kg). While mainly applicable to production-level aflatoxin screening methods with random and sporadic aflatoxin distribution in the samples, in the context of the present study this could provide insights into the increased CVs seen in the susceptible B73 line in EER and ISS shelling compared to WE shelling (Table 1).

Additionally, a recent review article was published advocating for increased research into standardizing sampling methods for aflatoxin in maize. While mainly focused on the industrial level, they highlighted the disadvantages of bulk kernel sampling such as the high variability of aflatoxin values between individual kernels [30]. This high level of variance in aflatoxin concentrations between individual kernels in bulk samples has been repeatedly observed over time and is well known in the aflatoxin research community. As an illustrative example, Johanssen et al. (2000) calculated that a 1.13 kg sample of maize with a 20 µg/kg aflatoxin concentration likely only contains six contaminated kernels per 10,000 kernels samples [31]. While this was mainly focused on industrial sampling, it highlights the challenge faced in obtaining representative samples for aflatoxin quantitation even in breeding programs. In the context of the current study, the proportion of kernels in the bulked samples actually inoculated with *A. flavus* and likely to contain aflatoxin is higher in EER and ISS shelling compared to WE shelling where sample dilution from excessive healthy kernel mass is possible. While these may reduce variance resulting from individual ear yield metrics like ear length and fill length, the increased proportion of inoculated kernels does have the potential to inflate aflatoxin measurements compared to WE shelling. For example, Mp719 exhibited significantly greater aflatoxin levels in ISS compared to WE and EER (Table 1). Therefore, while rank orders for the lines may be preserved, raw aflatoxin levels may vary between the methods, and EER may represent a compromise between avoiding excessive variance due to potential kernel loss during sample processing and overly concentrating aflatoxin levels due to a small sample size.

## 3. Conclusions

Variance in aflatoxin data is a consistent challenge for maize breeding programs. Here, we compared three different shelling techniques and evaluated the variance in aflatoxin data generated using these approaches for maize inbred germplasm and hybrids with contrasting susceptibility to aflatoxin contamination. In general, CVs were reduced when comparing WE shelling to EER and ISS shelling, though exceptions were observed. The variability seen in ISS shelling could be due to kernel retention differences between the lines and was greater in lines with higher overall aflatoxin levels. While EER shelling may not consistently produce the lowest CVs, our results indicate it can estimate aflatoxin contamination levels that do not significantly differ from WE shelling. Therefore, these reduced kernel mass shelling methods (EER and ISS) may provide some benefit in fine phenotyping of maize lines and may be useful in parental line evaluations and selections for breeding population development. However, the increased technical difficulty in performing these methods for a large number of ears may preclude their use in larger-scale evaluations.

## 4. Materials and Methods

### 4.1. Plant Cultivation and Design

For the comparison of different shelling methods and their effects on the variability of aflatoxin measurements, both inbred and hybrid germplasm were selected based on their previously characterized levels of resistance to aflatoxin contamination as well as commercial utility [9,32]. Three inbred lines B73 (S), SynAM1 P43 (MR), and Mp719 (R); and two hybrids SynAM1 P43 × B73 (F1 hybrid, unknown resistance) and DKC 67-44 (Dekalb/Bayer CropScience commercial F1 hybrid) were selected for the study. These lines were planted in two-row plots 5.1 m in length with a seeding rate of 25 seed per plot (15.2 cm (6 in) spacing) in a randomized complete block design with five replicate plots per line per shelling method (75 total plots) at the Mississippi State University R. R. Foil Plant Science Research Center, Starkville, MS, USA. After germination, ~1.0 m alleys were cut between plots. Standard agronomic practices were followed for all plots including fertilization and weed control. No fungicide applications were performed during the experiment.

### 4.2. Isolate Culturing and Inoculum Preparation

The *A. flavus* isolate AF13, a highly aflatoxigenic and stress tolerant isolate [33], was obtained from the USDA-ARS Crop Genetics and Breeding Research Unit, Tifton, GA, USA. The received isolate was transferred to V8 agar (20% V8 juice, 1% CaCO_3_, 3% agar), and stocks were generated by collecting five agar plugs from the growing edge of colonies in an amber vial containing 5 mL of sterile water. This was then used as inoculum to produce fresh cultures for field inoculum on V8 agar plates. Five-day-old cultures on V8 agar were flooded with ~20 mL of 0.1% (*v*/*v*) Tween 20 each and a sterile spreading loop was used to work the conidia into solution. The conidial suspensions were then collected and mixed by gentle shaking. The concentration of this suspension was then measured using a hemocytometer and adjusted to 4.0 × 10^6^ conidia/mL for use as inoculum in the field. Diluted inoculum was stored at 4 °C until used.

### 4.3. Inoculation and Harvesting

All plants were allowed to open pollinate during the experiment. Tassel and silk dates were recorded to determine pollination dates independently for each plot when at least half of the plants in the individual plot had tasseled or silked, respectively. At 14 ± 1 days after pollination (DAP), the plants were inoculated as previously described [9]. Briefly, five individual plants in each row within each plot were selected at random for inoculation with *A. flavus*. For inoculation, an Idico tree marking gun (Idico, New York, NY, USA) outfitted with an 18-gauge hypodermic needle was pressed through the husk and into each ear slightly off-center penetrating the husk and kernels on one side of the ear. Then, approximately 1.7 mL (two pumps of the gun) of *A. flavus* AF13 conidial suspension was injected into the ear. Following inoculation, the plants were labeled and allowed to grow under field conditions till maturity at 50 ± 1 days after inoculation (DAI, 65 ± 1 DAP). The five inoculated ears within each plot row were then bulk harvested to yield two bulk samples per plot (total of 150 samples) for shelling and aflatoxin analyses. The harvested ears were then placed into mesh bags and dried at ~51.7 °C (~125 °F) for 14 days prior to shelling and sample preparation.

### 4.4. Shelling and Sample Preparation

After harvesting and drying, the ears were shelled using one of three different methods (Figure 1). For whole ear (WE) shelling, all kernels on each inoculated ear were bulk shelled together using an SCS-2 corn sheller (Agriculex, Guelph, Ontario, Canada). For ear end removal (EER) shelling, non-inoculated kernels on the ends of each ear were removed to a level four kernel rows above and below the inoculation site/area with visible fungal growth. The use of four surrounding kernel rows was selected based on findings of King and Scott [34]. The remaining kernels on each ear were then bulk shelled using the SCS-2 corn sheller (Agriculex, Guelph, Ontario, Canada). For inoculation site-surrounding (ISS) shelling, only kernels damaged by inoculation and those immediately surrounding them were retained. All other non-inoculated kernels were manually removed. The remaining kernels on each ear were then manually bulk shelled. For all methods, the masses of kernels collected from each individual ear was recorded along with the length of each harvested ear (Tables S1 and S3). After shelling, the collected kernels from each plot row were then bulked together as a single grain sample. These samples were then thoroughly mixed and ground using a Romer Sample Mill (Series II, Romer Labs, Newark, DE, USA). These ground samples were then used for aflatoxin measurements.

### 4.5. Aflatoxin Quantification

Total aflatoxin for each sample was quantified using AflaTest column purification in conjunction with a VICAM Series 4 fluorometer according to the manufacturer’s instructions (VICAM, Milford, MA, USA). Although the AflaTest column can detect several aflatoxins (B, G, and M), *A. flavus* AF13 only produces B_1_ and B_2_ aflatoxins [33]. Briefly, a 50.0 g subsample of each sample was taken for aflatoxin quantification. In instances where a total sample mass was less than 50.0 g, calculations of final aflatoxin concentrations were adjusted accordingly. The subsample was combined with 5.0 g of sodium chloride and 100 mL of 80:20 methanol:water and placed into a blender. This was blended for one minute on high speed then filtered through fluted filter paper. After filtration, 10 mL of filtrate was then diluted with 40 mL of purified water in a clean beaker and mixed. This diluted extract was then filtered with a 1.5 µm glass microfiber filter. Following filtration, 2.0 mL of the diluted extract was passed through an AflaTest column. The column was then rinsed twice using 5.0 mL of purified water. Captured aflatoxins were then eluted from the column into a VICAM cuvette using 1.0 mL of HPLC-grade 100% methanol. For aflatoxin quantification, 1.0 mL of AflaTest developer solution was added to the eluted aflatoxin solution, mixed, and aflatoxins were quantified using the VICAM fluorometer. The VICAM fluorometer which was calibrated each day of use, per manual instructions, using manufacturer-provided aflatoxin standards. After calibrating but before sample testing, the methanol and water used for the extractions and dilutions were measured on the fluorometer for background fluorescence. Extracts with aflatoxin concentrations exceeding the assay range of detection (5.0–300 ppb, µg/kg) were diluted and remeasured, and the resultant measurements were multiplied by the dilution factor to determine the final concentration. All concentrations are reported in parts per billion (ppb, µg/kg).

### 4.6. Data Analysis

The obtained aflatoxin, sample mass, and ear length data was analyzed using R (v 4.3.0) and R Studio (v 2023.03.1). For aflatoxin data, the data were subjected to removal of outliers and log_2_(x + 1) transformation prior to an analysis of variance (ANOVA) to yield more normal distributions to satisfy analytical model assumptions (Figure S3). Outliers were determined from the raw aflatoxin data based on the interquartile range (IQR); the same method used by the R package ggplot2 for visualizations and excluded (Figure S2). An ANOVA coupled with Tukey’s post-hoc analysis was performed to examine treatment and variety effects on the data. Coefficients of variance (CVs) were calculated by dividing the standard deviation of the data by the arithmetic mean of the data for each line × shelling method combination, then multiplying the result by 100. The modified signed-likelihood ratio test (modified SLRT) for equality of CVs was used to test significant differences in variation of raw and transformed aflatoxin data [20]. Statistical tests that do not have assumptions of normal data distribution, such as the Kruskal–Wallis test and Dunn’s test, were used when appropriate [35,36,37,38]. Raw and transformed data are provided in the Supplemental File (File S1).

## Figures and Tables

**Table 1:** Two-Way Factorial Analysis of Variance of Transformed ^1^ Aflatoxin Data.

Factor	Df	Sums of Squares	Mean Sums of Squares	F-Value	*p*-Value	
Line	4	391.3	97.82	120.7	<2.00 × 10^−16^	***
Treatment	2	3.1	1.57	1.94	0.15	
Rep	4	1.6	0.41	0.51	0.73	
Line:Treatment	8	66	8.25	10.2	6.71 × 10^−16^	***
Line:Treatment:Rep	55	41.7	0.76	0.94	0.6	
Residuals	61	49.4	0.81			

^1^ Raw aflatoxin measurements were subjected to log_2_(x + 1) transformation to ensure normal data distributions. Significant at *p* < 0.001 ***.

**Table 2:** Raw Aflatoxin Measurements, by Shelling Method.

	Method 1—Whole Ear (WE) Shelling					
Line	Trait	HSD	CV	Q1	Q3	IQR
*Inbreds*						
B73	12,000 ± 2062	a	17.2	11,100	12,000	900
SynAM1 P43	774 ± 733	e	94.6	308	670	362
Mp719	84 ± 50	f	59.6	39	110	71
*Hybrids*						
SynAM1 P43 × B73	1448 ± 1100	bcde	75.9	730	2090	1360
DKC 67-44	2112 ± 1300	bc	61.6	1260	2900	1640
	**Method 2—Ear End Removal** (**EER**) **Shelling**					
**Line**	**Trait**	**HSD**	**CV**	**Q1**	**Q3**	**IQR**
*Inbreds*						
B73	12,064 ± 6414	a	53.2	7200	16,400	9200
SynAM1 P43	840 ± 675	de	80.4	480	1100	620
Mp719	199 ± 122	f	61.5	110	310	200
*Hybrids*						
SynAM1 P43 × B73	1400 ± 746	bcde	53.3	860	1890	1030
DKC 67-44	1658 ± 585	bcd	35.3	1120	1960	840
	**Method 3—Inoculation Site-Surrounding** (**ISS**) **Shelling**					
**Line**	**Trait**	**HSD**	**CV**	**Q1**	**Q3**	**IQR**
*Inbreds*						
B73	3440 ± 2322	b	67.5	1840	4400	2560
SynAM1 P43	1188 ± 267	bcde	22.5	1065	1350	285
Mp719	559 ± 269	e	48.2	328	780	453
*Hybrids*						
SynAM1 P43 × B73	928 ± 258	cde	27.8	790	945	155
DKC 67-44	2194 ± 930	bc	42.4	1520	2800	1280
	**Methods Combined**					
**Line**	**Trait**	**HSD**	**CV**	**Q1**	**Q3**	**IQR**
*Inbreds*						
B73	9170 ± 5839	a	63.7	4400	12,000	7600
SynAM1 P43	924 ± 609	c	65.9	493	1310	818
Mp719	291 ± 270	d	93	92.5	380	288
*Hybrids*						
SynAM1 P43 × B73	1282 ± 811	bc	63.3	760	1610	850
DKC 67-44	1977 ± 991	b	50.1	1260	2300	1040

Aflatoxin measurements (µg/kg) are raw averages from ten replicates ± standard deviation. CV—coefficient of variance (100 × (standard deviation/mean for all reps for a given line)), Q1—lower quartile, Q3—upper quartile, IQR—interquartile range. Measurements within a trait possessing the same letter labels were not significantly different based on Tukey’s post-hoc analysis (HSD, α = 0.05). Analytical results for aflatoxin measurements are based on log_2_(x + 1) transformed data. Raw outliers identified in Figure S2 were removed. Lines are listed as inbreds first, followed by hybrids.

**Table 3:** Modified Signed-Likelihood Ratio Test (SLRT) for Equality of Coefficients of Variance for Raw and Transformed Aflatoxin Data.

	Test Statistic		*p*-Value ^2^	
	Raw	Transformed ^1^	Raw	Transformed ^1^
***Overall Data Level***				
Line Effect	18.45	37.84	0.001 **	1.2 × 10^−7^ ***
Shelling Effect	1.12	24.07	0.6	5. × 10^−6^ ***
Line × Shelling Effect	29.28	53.14	0.010 **	1.8 × 10^−6^ ***
***Shelling Effect by Line***				
B73	6.49	15.03	0.04 *	0.0005 ***
SynAM1 P43	3.52	9.91	0.2	0.007 **
Mp719	6.21	3.07	0.045 *	0.22
SynAM1 P43 × B73	1.13	9.42	0.57	0.009 **
DKC 67-44	1.47	1.54	0.48	0.46
***Pairwise Comparisons* ^3^**				
B73 WE v. ERR	2.99	12.69	0.084	0.0004 ***
B73 WE v. ISS	6.41	15.25	0.01 *	9.4 × 10^−5^ ***
B73 EER v. ISS	0.82	0.37	0.37	0.54
P43 WE v. EER	0.09	0.03	0.77	0.85
P43 WE v. ISS	8.21	9.26	0.004 **	0.002 **
P43 EER v. ISS	6.63	8.56	0.01 *	0.003 **
Mp719 WE v. ERR	1.97	0.14	0.16	0.71
Mp719 WE v. ISS	1.24	2.99	0.27	0.084
Mp719 EER v. ISS	5.81	1.88	0.02 *	0.17
P43 × B73 WE v. EER	0.62	2.44	0.43	0.12
P43 × B73 WE v. ISS	4.60	8.46	0.03 *	0.004 **
P43 × B73 EER v. ISS	2.32	3.12	0.13	0.08
DKC 67-44 WE v. EER	1.72	1.46	0.19	0.23
DKC 67-44 WE v. ISS	0.69	0.49	0.41	0.48
DKC 67-44 EER v. ISS	0.12	0.15	0.72	0.70

Analysis of significant differences in coefficients of variance (CV) based on Krishnamoorthy and Lee [20]. Factors for this analysis are listed in bold italics. ^1^ Raw aflatoxin measurements were subjected to log_2_(x + 1) transformation and removal of outliers to ensure normal data distributions. ^2^ Significant at *p* < 0.05 *, *p* < 0.01 **, and *p* < 0.001 ***. ^3^ The “SynAM1” of P43 was removed for simplicity.

## Data Availability

Fungal isolates are available upon request by contacting the corresponding author.

## Supplementary Materials

The following supporting information can be downloaded at: https://www.mdpi.com/article/10.3390/toxins16070324/s1, Figure S1: Example of kernel and ear size variation within a replicate plot inoculated with *Aspergillus flavus* strain AF13 for each of the shelling methods; Figure S2: Boxplots of raw aflatoxin levels (µg/kg) for each line per each shelling method; Figure S3: Histogram of raw and log_2_(x + 1) transformed aflatoxin levels (µg/kg) for each shelling method; Table S1: Shelled Kernel Mass Measurements by Shelling Method; Table S2: Analysis of Average Shelled Kernel Mass; Table S3: Total and Filled Ear Lengths Measured for Each Line; Table S4: Two-Way Factorial Analyses of Variance of Ear Length and Filling; File S1: Raw and transformed phenotypic data.
