# Supplementary material for: Effects of Different Shelling Methods on Data Variability during Field Screening for Reduced Aflatoxin Contamination in Maize
Source: Toxins (Basel). 2024 Jul 19;16(7):324. doi: 10.3390/toxins16070324 (PMC11281036; doi:10.3390/toxins16070324)
Supplement: Supplementary file 1 [file toxins-16-00324-s001.zip › Supplemental Materials.pdf]

Supplementary Materials: Effects of different shelling methods on data variability during field screening for reduced aflatoxin contamination in maize

Table S1. Shelled Kernel Mass Measurements by Shelling Method

	Method 1 - Whole Ear Shelling (WE)							
**Line**	**Trait**				**CV**	**Q1**	**Q3**	**IQR**
Inbreds								
**B73**	56.8	±	17.3	d	30.35	47.0	66.0	19.0
**SynAM1 P43**	105.0	±	21.9	c	20.90	91.5	116.8	25.3
**Mp719**	53.5	±	9.4	d	17.52	48.0	58.8	10.8
Hybrids								
**SynAM1 P43 x B73**	142.2	±	20.9	b	14.69	128.0	151.0	23.0
**DKC 67-44**	183.6	±	33.0	a	17.92	170.0	202.8	32.8
	**Method 2 - Ear End Removal Shelling (EER)**							
**Line**	**Trait**				**CV**	**Q1**	**Q3**	**IQR**
Inbreds								
**B73**	23.3	±	5.8	f	24.72	19.2	27.1	8.0
**SynAM1 P43**	35.5	±	9.3	e	26.09	30.5	40.5	10.0
**Mp719**	20.6	±	4.5	f	21.91	18.5	23.5	5.0
Hybrids								
**SynAM1 P43 x B73**	35.9	±	6.3	e	17.46	32.3	40.9	8.5
**DKC 67-44**	50.1	±	7.5	d	14.91	45.6	54.8	9.2
	**Method 3 - Inoculation Site-Surrounding Shelling (ISS)**							
**Line**	**Trait**				**CV**	**Q1**	**Q3**	**IQR**
Inbreds								
**B73**	3.2	±	1.3	g	41.49	2.2	3.8	1.6
**SynAM1 P43**	4.3	±	1.0	g	22.45	3.8	4.8	1.0
**Mp719**	2.9	±	0.7	g	25.09	2.4	3.3	0.9
Hybrids								
**SynAM1 P43 x B73**	4.3	±	1.0	g	22.38	3.7	5.0	1.3
**DKC 67-44**	6.1	±	1.3	g	20.80	5.3	6.8	1.6

Shelled kernel masses are average measurements of five pooled ears from ten replicate plots in grams (g) ± standard deviation. CV - Coefficient of Variance (100x(standard deviation / mean for all reps for a given line)), Q1 - Lower Quartile, Q3 - Upper Quartile, IQR - Interquartile Range. Measurements within a trait possessing the same letter labels were not significantly different based on Tukey's Post-hoc Analysis (α = 0.05). The shelled kernel mass analysis was based on two-way factorial ANOVA with the assumption of normal data distribution.

Table S2. Analysis of Average Shelled Kernel Mass

			**Kruskal-Wallis Rank Sum Test**[1]		
**Model**	**Df**	**X^2**	**P-Value**		
**Mass ~ Treatment**	2	127.32	< 2.2E-16	***	
**Mass ~ Line**	4	18.52	< 2.2E-16	***	
**Mass ~ Rep**	4	0.044	0.9998		
**Mass ~ Row**	1	0.02	0.8887		
			**Dunn's Test for Multiple Comparisons**[2]		
**Comparison**		**Z**	**Unadjusted P-Value**	**Adjusted P-Value**	
By Treatment					
**EER v. ISS**		5.80	6.55E-09	1.96E-08	***
**EER v. WE**		-5.54	3.06E-08	9.18E-08	***
**ISS v. WE**		-11.3	1.59E-29	4.76E-29	***
Pairwise Comparisons[3]					
**B73 ISS v. B73 WE**		-4.87	1.09E-06	1.15E-04	***
**B73 ISS v. DKC 67-44 EER**		-4.47	8.00E-06	8.40E-04	***
**B73 EER v. DKC 67-44 WE**		-4.29	1.78E-05	1.87E-03	**
**B73 ISS v. DKC 67-44 WE**		-6.77	1.28E-11	1.35E-09	***
**DKC 67-44 ISS v. DKC 67-44 WE**		-4.89	9.92E-07	1.04E-04	***
**DKC 67-44 WE v. Mp719 EER**		4.55	5.34E-06	5.60E-04	***
**B73 WE v. Mp719 ISS**		5.09	3.56E-07	3.74E-05	***
**DKC 67-44 EER v. Mp719 ISS**		4.68	2.85E-06	2.99E-04	***
**DKC 67-44 WE v. Mp719 ISS**		6.99	2.81E-12	2.95E-10	***
**B73 ISS v. Mp719 WE**		-4.69	2.78E-06	2.91E-04	***
**Mp719 ISS v. Mp719 WE**		-4.9	9.43E-07	9.90E-05	***
**Mp719 ISS v. P43 EER**		-3.59	3.29E-04	3.45E-02	*
**B73 WE v. P43 ISS**		4.07	4.73E-05	4.97E-03	**
**DKC 67-44 EER v. P43 ISS**		3.66	2.53E-04	2.66E-02	*
**DKC 67-44 WE v. P43 ISS**		5.96	2.45E-09	2.57E-07	***
**Mp719 WE v. P43 ISS**		3.88	1.04E-04	1.09E-02	*
**B73 ISS v. P43 WE**		-5.73	1.02E-08	1.07E-06	***
**DKC 67-44 ISS v. P43 WE**		-3.91	9.24E-05	9.70E-03	**
**Mp719 EER v. P43 WE**		-3.51	4.52E-04	4.74E-02	*
**Mp719 ISS v. P43 WE**		-5.94	2.78E-09	2.92E-07	***
**P43 ISS v. P43 WE**		-4.92	8.59E-07	9.02E-05	***
**Mp719 ISS v. P43 x B73 EER**		-3.6	3.16E-04	3.32E-02	*
**B73 WE v. P43 x B73 ISS**		4.02	5.71E-05	6.00E-03	**
**DKC 67-44 EER v. P43 x B73 ISS**		3.61	3.01E-04	3.16E-02	*
**DKC 67-44 WE v. P43 x B73 ISS**		5.92	3.21E-09	3.37E-07	***

**Mp719 WE v. P43 x B73 ISS**	3.84	1.25E-04	1.31E-02	*
**P43 WE v. P43 x B73 ISS**	4.88	1.08E-06	1.13E-04	***
**B73 EER v. P43 x B73 WE**	-3.77	1.64E-04	1.72E-02	***
**B73 ISS v. P43 x B73 WE**	-6.25	4.13E-10	4.34E-08	***
**DKC 67-44 ISS v. P43 x B73 WE**	-4.4	1.07E-05	1.13E-03	**
**Mp719 EER v. P43 x B73 WE**	-4.03	5.59E-05	5.87E-03	**
**Mp719 ISS v. P43 x B73 WE**	-6.47	1.01E-10	1.06E-08	***
**P43 ISS v. P43 x B73 WE**	-5.44	5.24E-08	5.50E-06	***
**P43 x B73 ISS v. P43 x B73 WE**	-5.4	6.71E-08	7.04E-06	***

Two-Way Analysis of Variance[4]

**Factor**	**Df**	**Sums of Squares**	**Mean Sums of Squares**	**F-Value**	**P-Value**	
**Line**	4	70,758	17,689	449.057	< 2.0E-16	***
**Treatment**	2	279,133	139,566	3542.979	< 2.0E-16	***
**Rep**	4	33	8	0.208	0.933	
**Row**	1	35	35	0.899	0.346	
**Line:Treatment**	8	65,781	8223	208.736	< 2.0E-16	***
**Line:Treatment:Rep**	55	2227	40	1.028	0.452	
**Residuals**	73	2876	39			

[1]Kruskal-Wallis test allows for analysis of variance without the assumption of a normal data distribution. [2]Dunn's test allows for multiple comparisons. It also does not have the assumption of normal data distributions. P-values were adjusted using the Bonferroni method. [3]Of the 105 possible pairwise comparisons, only the pairs with adjusted p-values < 0.05 are shown. The "SynAM1" of P43 was removed for simplicity. [4]Two-way ANOVA performed on raw data. Results may overestimate significance due to non-normal distribution. However, results generally agree with the Kruskal-Wallis and Dunn tests. Significant at P < 0.05 *, P < 0.01 **, and P < 0.001 ***.

Table S3. Total and Filled Ear Lengths Measured for Each Line

Line	Total Ear Length (mm)							
	Trait				CV	Q1	Q3	IQR
Inbreds								
**B73**	135.4	±	11.5	e	8.46	129.0	142.0	13.0
**SynAM1 P43**	162.7	±	16.1	c	9.89	151.0	172.0	21.0
**Mp719**	144.1	±	13.8	d	9.61	137.0	152.0	15.0
Hybrids								
**SynAM1 P43 x B73**	179.8	±	14.1	b	7.86	170.0	189.5	19.5
**DKC 67-44**	190.7	±	13.1	a	6.85	183.0	200.5	17.5

Line	Kernel Filled Ear Length (mm)							
	Trait				CV	Q1	Q3	IQR
Inbreds								
**B73**	122.4	±	13.1	e	10.71	115.0	132.0	17.0
**SynAM1 P43**	149.6	±	17.2	c	11.49	138.0	160.0	22.0
**Mp719**	132.3	±	14.0	d	10.60	126.0	140.0	14.0
Hybrids								
**SynAM1 P43 x B73**	169.2	±	15.5	b	9.16	161.0	180.0	19.0
**DKC 67-44**	181.3	±	15.5	a	8.54	175.0	190.5	15.5

Line	Percent Ear Fill (%)							
	Trait				CV	Q1	Q3	IQR
Inbreds								
**B73**	90.4	±	6.2	b	6.90	86.2	95.0	8.8
**SynAM1 P43**	92.0	±	5.5	b	5.92	89.5	95.6	6.1
**Mp719**	91.9	±	6.0	b	6.50	89.2	95.8	6.7
Hybrids								
**SynAM1 P43 x B73**	94.2	±	5.2	a	5.55	92.6	97.0	4.4
**DKC 67-44**	95.1	±	5.3	a	5.61	93.4	98.9	5.5

Each line had a max of 150 ears. Measurements within a trait possessing the same letter labels were not significantly different based on Tukey's Post-hoc Analysis (α = 0.05). CV - Coefficient of Variance (100x(standard deviation / mean for all reps for a given line)), Q1 - Lower Quartile, Q3 - Upper Quartile, IQR - Interquartile Range.

Table S4. Two-Way Factorial Analyses of Variance of Ear Length and Filling

Factor	Df	Sums of Squares	Mean Sums of Squares	F-Value	P-Value	
Ear Length						
**Line**	4	306,876	76,719	422.46	<2.00E-16	***
**Treatment**	2	3,017	1,508	8.31	0.0003	***
**Rep**	4	1,096	274	1.51	0.2	
**Row**	1	1	1	0.004	0.95	
**Line:Treatment**	8	1,936	242	1.33	0.22	
**Line:Treatment:Rep**	54	13,699	254	1.40	0.04	*
**Residuals**	648	117,676	182			
Ear Fill Length						
**Line**	4	342,193	85,548	379.44	<2.00E-16	***
**Treatment**	2	150	75	0.33	0.72	
**Rep**	4	934	234	1.04	0.39	
**Row**	1	10	10	0.04	0.84	
**Line:Treatment**	8	1,239	155	0.69	0.7	
**Line:Treatment:Rep**	54	16,124	299	1.32	0.07	
**Residuals**	648	146,096	225			

Significant at P < 0.05 *, P < 0.01 **, and P < 0.001 ***.

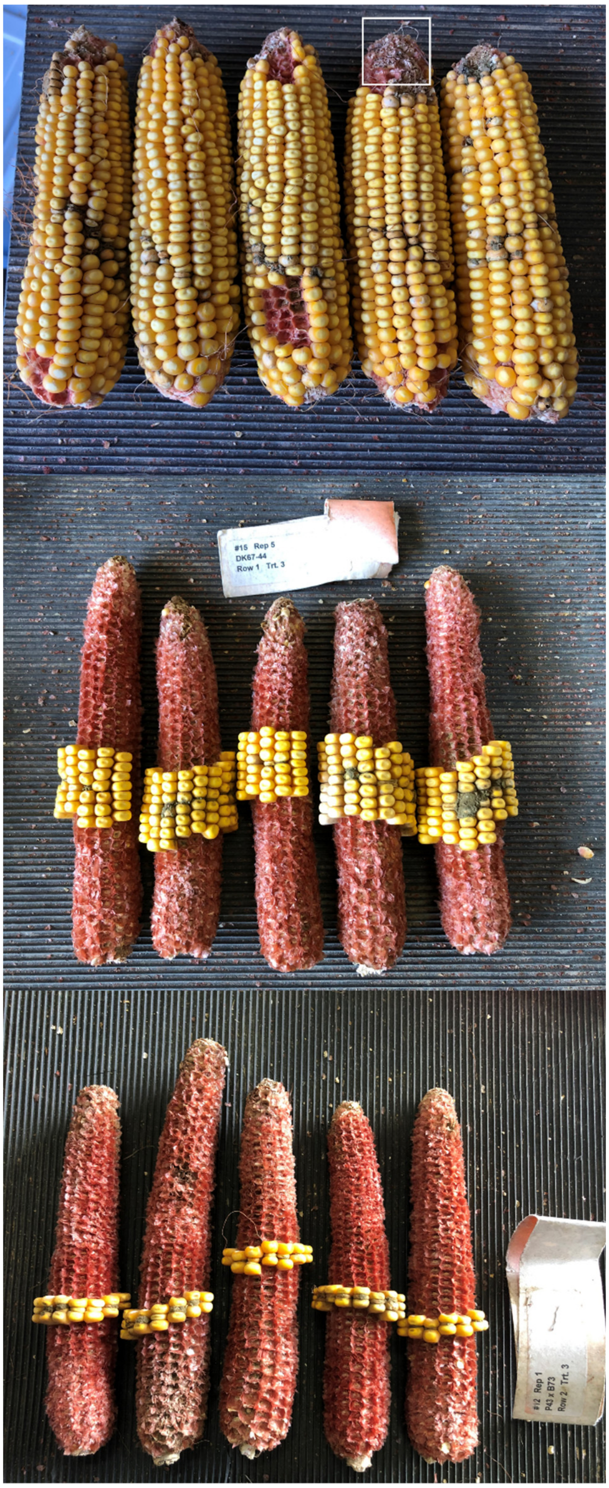

Figure S1. Example of kernel and ear size variation within a replicate plot inoculated with *Aspergillus flavus* strain AF13 for each of the shelling methods- whole ear shelling (WE, top), ear end removal shelling (EER, middle), and inoculation site-surrounding shelling (ISS, bottom). White box indicates example of typical ear damage (i.e., insect and improper filling).

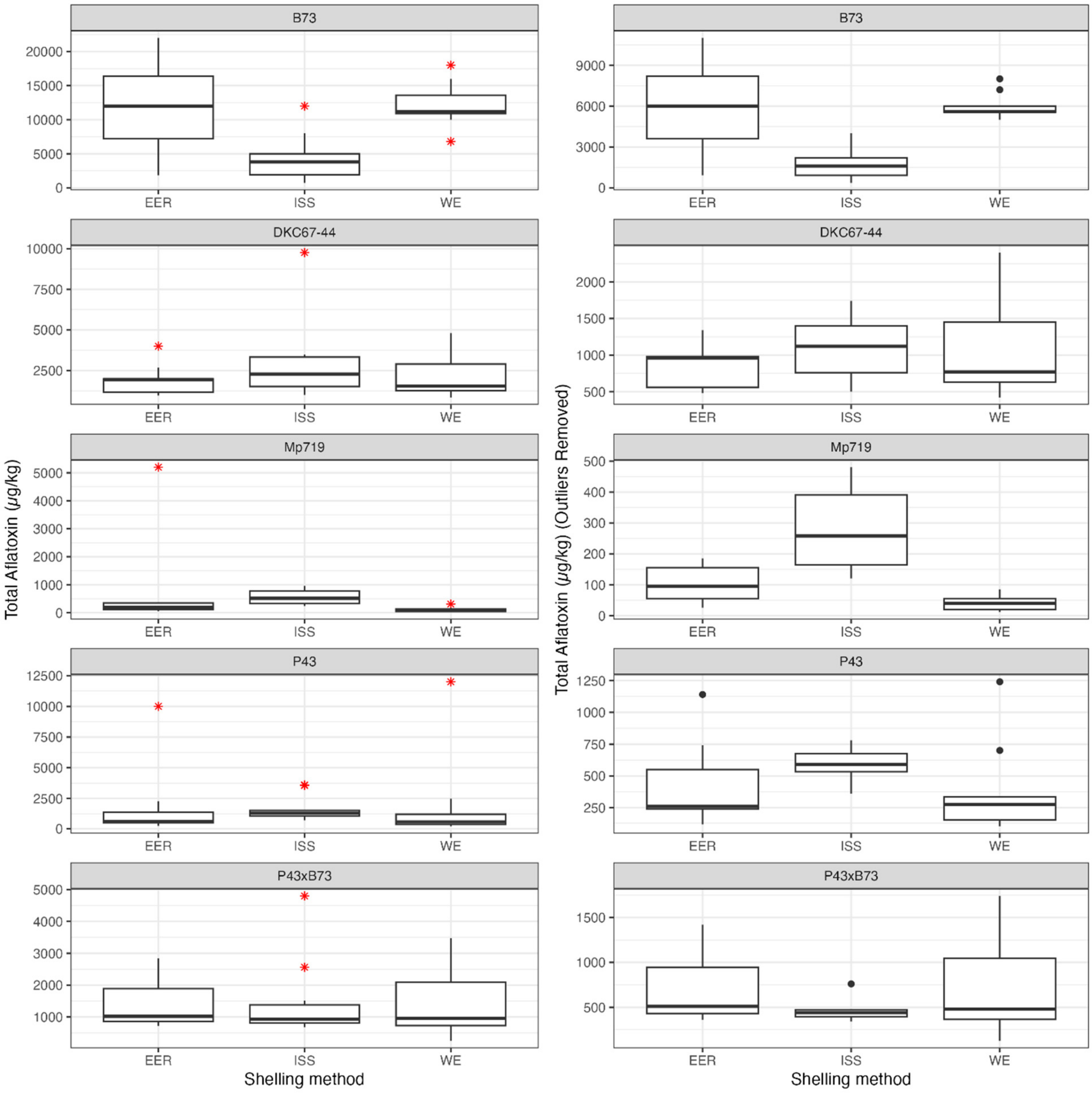

Figure S2. Boxplots of raw aflatoxin levels (μg/kg) for each line per each shelling method. Outliers identified by red asterisks (left) were removed from the raw data (right) before $\log_2(x+1)$ transformed. WE is whole ear shelling, EER is ear end removal shelling, and ISS is inoculation site-surrounding shelling. The "SynAM1" of P43 was removed for simplicity.

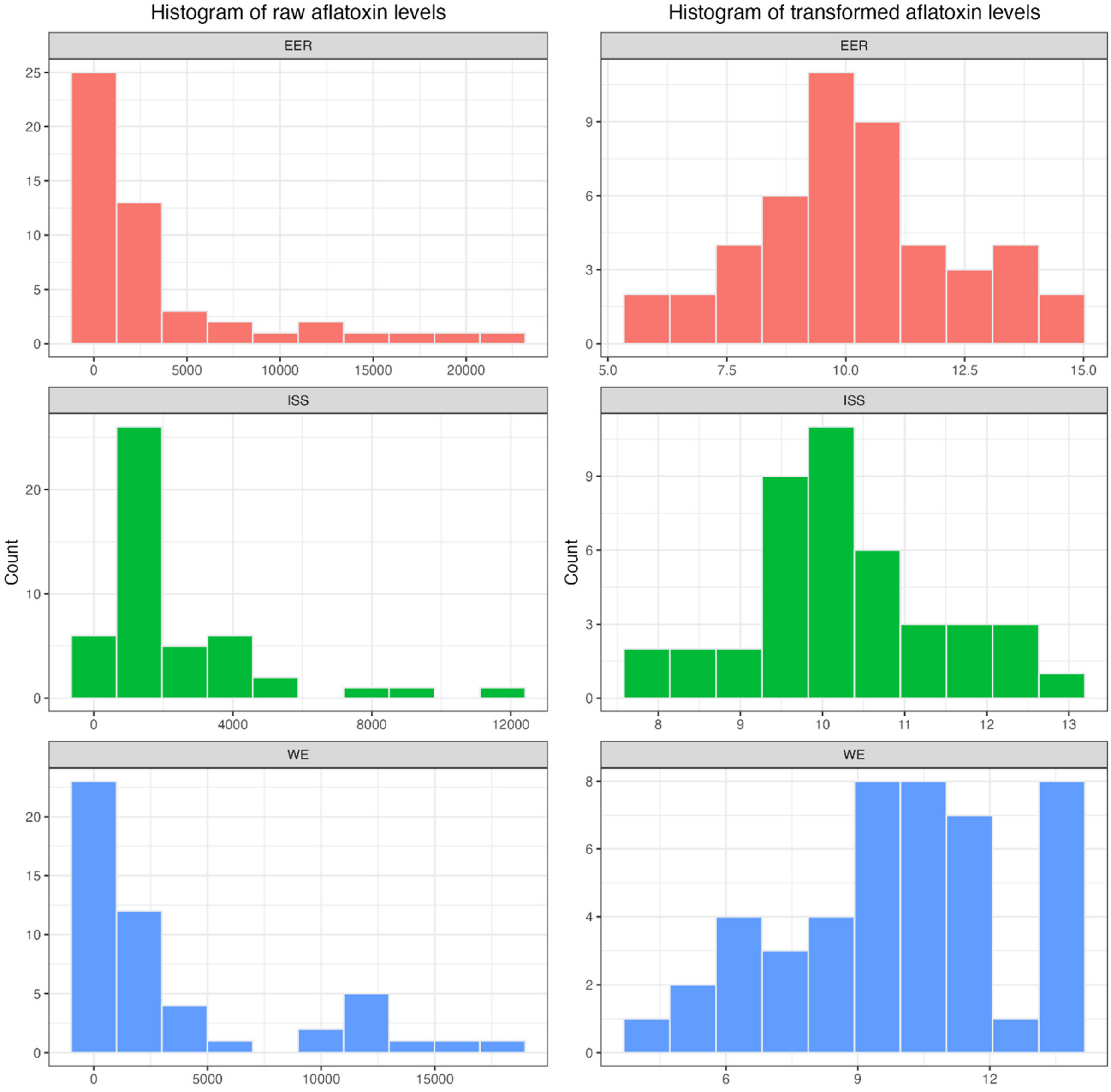

Figure S3. Histogram of raw and log2(x+1) transformed aflatoxin levels (μg/kg) for each shelling method. Outliers from the raw data are excluded in the transformation and 10 bins were used for the histogram. WE is whole ear shelling, EER is ear end removal shelling, and ISS is inoculation site-surrounding shelling.
